# Validity and Reliability of the Transcultural Arabic Adaptation of the Food-Mood Questionnaire Among College Students

**DOI:** 10.3390/ijerph21111509

**Published:** 2024-11-13

**Authors:** Lina Begdache, Hadia Radwan, Salma Abu Qiyas, Nada Abbas, Farah Naja

**Affiliations:** 1 Health and Wellness Studies Department, Binghamton University, Binghamton, NY 13902, USA; lina@binghamton.edu; 2 Department of Clinical Nutrition and Dietetics, Research Institute of Medical and Health Sciences, College of Health Sciences, University of Sharjah, Sharjah P.O. Box 27272, United Arab Emirates; hradwan@sharjah.ac.ae (H.R.); sabuqiyas@sharjah.ac.ae (S.A.Q.); abbas_nada@hotmail.com (N.A.); 3 Nutrition and Food Sciences Department, American University of Beirut, Beirut P.O. Box 11-0236, Lebanon

**Keywords:** mental health, food intake, youth, validation, United Arab Emirates

## Abstract

A culturally adapted screening tool for mental health and dietary quality is needed to address the significant challenges in mental health and suboptimal diets among college students. The purpose of this study was to validate the Food-Mood Questionnaire (FMQ), originally developed in English, among Arab college students. Students attending the University of Sharjah were invited to complete the questionnaire (*n* = 224). Two weeks later, participants completed the same questionnaire again. An exploratory factor analysis revealed three main factors: mental distress, prudent and Western diets. Cronbach’s α was 0.86, 0.72, and 0.531 for the three factors, respectively. The Intra-Class-Correlation (ICC) for the test–retest reliability ranged from 0.67 to 0.87 (*p* < 0.001). The findings of this study showed that the Arabic version of the FMQ is a valid and reliable tool and could be used to screen for the mental distress and dietary intake of college students in the Arab world.

## 1. Introduction

In the last couple of decades, mental disorders remained among the top ten leading causes of disease burden [1]. While these conditions pose a significant challenge across the entire lifespan, most mental disorders have their peak incidence during the transition from childhood to young adulthood, with about one in five people experiencing clinically relevant mental health problems before the age of 25 years [2]. According to the World Mental (WHO) Health Surveys International College Student Project, one-fifth of college students experienced mental disorders that lasted for at least 12 months [1]. College students experience high levels of mental distress, mostly due to academic pressures, financial stress, and uncertainties associated with future employment [3]. Mental health is a broad term that encompasses the emotional, psychological, and social dimensions of one’s life [4]. In this manuscript, mental health/distress refers to non-specific psychological distress. 

In addition to genetic predisposition, environmental exposures affect brain maturation and mental health during adolescence (ages 10–24 years), whereby diet quality has recently emerged as a significant modifiable factor. Several nutrients play a significant role in many aspects of brain function and health, such as neurotransmission, homeostasis, and structural support [5,6]. Low-quality foods, such as ultra-processed or packaged food, lack several crucial nutrients to support brain function, such as omega-3 fats, complex carbohydrates, and B vitamins [7]. On the other hand, nutrient-dense diets, such as the Mediterranean diet, were shown to have a positive effect on improving mental well-being and reducing the risk of mental disorders [8]. College students are particularly prone to the consumption of suboptimal diets [9,10]. Students are often faced with busy schedules, leaving them little time for meal preparation. Additionally, limited budgets may lead them to prioritize cheap, convenient, but often unhealthy food options. Moreover, the stress and pressure of academic life can contribute to emotional eating or a reliance on fast food and processed snacks for quick energy boosts [11]. College students’ poor mental health, coupled with their suboptimal diet, constitutes a vicious circle; therefore, screening and identifying mental health disorders and unhealthy eating behaviors is critical for the development of effective public health interventions.

In many Arab countries, the burden of mental health is amplified given the stigmatization of mental disorders and the associated sense of shame, mostly due to cultural and social norms [12]. The United Arab Emirates (UAE), an oil-rich Arab country in the Arabian Gulf, reported soaring prevalence rates of depression, anxiety, and stress, reaching as high as 38% [13]. Coupled with this heavy mental health burden, the UAE has been experiencing a rapid shift in dietary patterns from traditional foods rich in dairy products, vegetables, and fruits to westernized energy-dense foods rich in saturated fat [14]. The double burden of mental disorders and unhealthy eating is particularly significant among young adults, who make up thirty-four percent of the total UAE population [15]. Screening strategies, early detection, and interventions among youth may allow for more effective public health programs in the country. In this context, a culturally adapted tool to screen for mental health as well as dietary quality is needed [16,17].

The Food-Mood Questionnaire (FMQ) developed by Begdache et al., 2019 [18], is a short-validated tool that is useful in screening college students’ mental health and dietary quality [19,20]. The FMQ was originally developed and validated in English and was later translated and validated in Turkish [21]. To date, there exists no Arabic version of FMQ. Therefore, the study aims to examine the validity and reliability of an adapted Arabic FMQ among college students. The main hypothesis in this study is that the adapted Arabic FMQ is a valid and reliable tool to examine both the mental health and dietary intake among college students.

## 2. Materials and Methods

### 2.1. Study Design and Participants

The study was conducted according to the guidelines laid down in the Declaration of Helsinki and all procedures involving human subjects were approved by the Institutional Review Board at the University of Sharjah (REC-21-04-04-01). Written informed consent was obtained from all study participants. This cross-sectional study was conducted between May 2021 and July 2021. The study population consisted of students attending the University of Sharjah, United Arab Emirates. The inclusion criteria were as follows: (1) adults 18 years or older, (2) enrolled as a student at the university, and (3) using the Arabic language for communication. Participants completed a multi-component questionnaire using Google Forms. The link to the questionnaire was sent by the central administration to all students via university emails. Participants read and signed an informed consent that included information about the purpose of the study, its protocol, and the time needed to complete the questionnaire. The anonymity and voluntary nature of participation were also stated in the consent form. All students who completed the questionnaire were invited to complete it again after a period of two weeks for the test and retest analysis.

### 2.2. The Food and Mood Questionnaire (FMQ)

The FMQ is a multi-component questionnaire that consists of three main sections. The first section included questions related to the sociodemographic characteristics of participants, such as age, gender, marital status, nationality, living place, major of study, family income, and academic year. The second section included questions related to lifestyle and addressed smoking (smoker, non-smoker) and dietary patterns. For the latter, participants were asked how they perceived their dietary pattern and were given two choices: Healthy, rich in fruits, vegetables, and whole grains; and Unhealthy: rich in refined grains, red meat, and solid fats. The last section comprised the FMQ, which consisted of 22 questions, distributed as 6 questions focusing on mental health and 16 on food consumption. Details about the questions in the FMQ are described elsewhere [18]. Briefly, the mental health section consists of the Kessler K6 non-specific distress scale, which is widely used as a screening tool for mental illness [22]. Participants were asked to refer to the last 12 months and report on six main symptoms: whether they felt nervous, restless or fidgety, felt worthless, felt depressed, or felt that everything was an effort. The frequency of these symptoms included ‘none of the time’, ‘a little of the time’, ‘some of the time’, ‘most of the time’, or ‘all of the time’. For food consumption, 13 food/food group items were included in the questionnaire. These items were selected given their significant mental health implications, especially in this age group. For each item, participants were asked about the frequency of weekly consumption (none, 1 time, 2 times, 3 times, 4 times, 5 times, or more). The dietary assessment in the FMQ is intended to provide a simple yet broad overview of dietary intake. With a low participation burden, this approach reduces the variation in responses due to differences in portion size estimation, and hence allows for comparison between groups and populations [23]. In addition to the food and food group items, three questions addressing exercise, breakfast consumption, and supplements use were included in the FMQ. Permission to translate the FMQ was obtained from Begdache et al. [18]. The English version of the FMQ was translated into Arabic by two native speakers. To ensure a parallel form of reliability, a forward–backward translation method was used. With the forward translation, a translator in fluent English translated the questionnaire into Arabic. Then, the Arabic version was back-translated into English. The original and back-translated English versions were then compared for accuracy.

A group of three nutritionists qualitatively assessed the content and face validity of the translated FMQ. The experts revised the Arabic questionnaire for simplicity, cultural relevance, and ease. The nutrition experts evaluated the extent to which the Arabic FMQ represents all the food groups commonly consumed in the country, as well as the use of appropriate syntax to promote the readability of the questions. The translated FMQ was pilot-tested on 20 students, and their feedback was used to further improve the structure of the questions. Data from the pilot test phase were not included in the analysis for this study. The questionnaire used in this study is found in Supplementary Materials.

### 2.3. Statistical Analysis

The baseline characteristics of the study participants were presented as frequency and percentage for categorical variables and mean ± SD for continuous variables. The construct validity of the questionnaire was examined using the exploratory factor analysis (EFA) and confirmatory factor analysis (CFA), which were performed to identify and confirm the scale dimensions. A scree plot with 20 simulations was generated to assess scale dimensionality. The number of components retained was based on the “parallel analysis” approach. The latter is a technique that compares the scree of factors of the observed data with that of a random data matrix of the same size as the original and identifies the number of factors that are less likely to be due to chance [24]. The concurrent validity of the questionnaire was examined by investigating the association between the identified dietary component scores and the perceived type of dietary patterns reported by the participants, using simple and multiple linear regression analyses. For the internal validity of the questionnaire, the Cronbach alpha coefficients of each component (factor) were examined. In addition, the item–total score correlation, inter-item correlation, and Cronbach alpha if the item was deleted were also reported. The test–retest reliability of the questionnaire was evaluated using Intra Class Correlation (ICC). In this study, no scoring was used in the evaluation of the responses. Each item in the questionnaire was treated individually in the statistical analysis. All analyses were performed using R version 4.0.1.

## 3. Results

Around 2000 students who were eligible to participate (according to the inclusion criteria in this study) were invited by email. Of those, 224 took part in the study (11%). The baseline characteristics of the study population are summarized in Table 1. The mean age of the study population was 20.7 ± 4.1 years, and the majority were females (72.8%) and single (89.7%). More than half of the study population were Emiratis (61.2%), 35.3% were Arabs (non-Emirati), and 3.6% were Asian/African. One in three participants was a resident of Sharjah (33%). The distribution of the students’ majors was as follows: medicine and health (43.8%), sciences (40.6%), engineering (11.2%), and arts and humanities (4.5%). Family income for two-thirds of the study population was more than AED 10,000 (64.3%), and the majority of participants were non-smokers (83.9%). When asked about their dietary pattern, only 15.2% reported following a healthy pattern, defined as a diet rich in fruits, vegetables, and whole grains, while the majority (84.8%) classified their diet as unhealthy (rich in refined grains, red meat, and solid fats).

**Figure 1:**
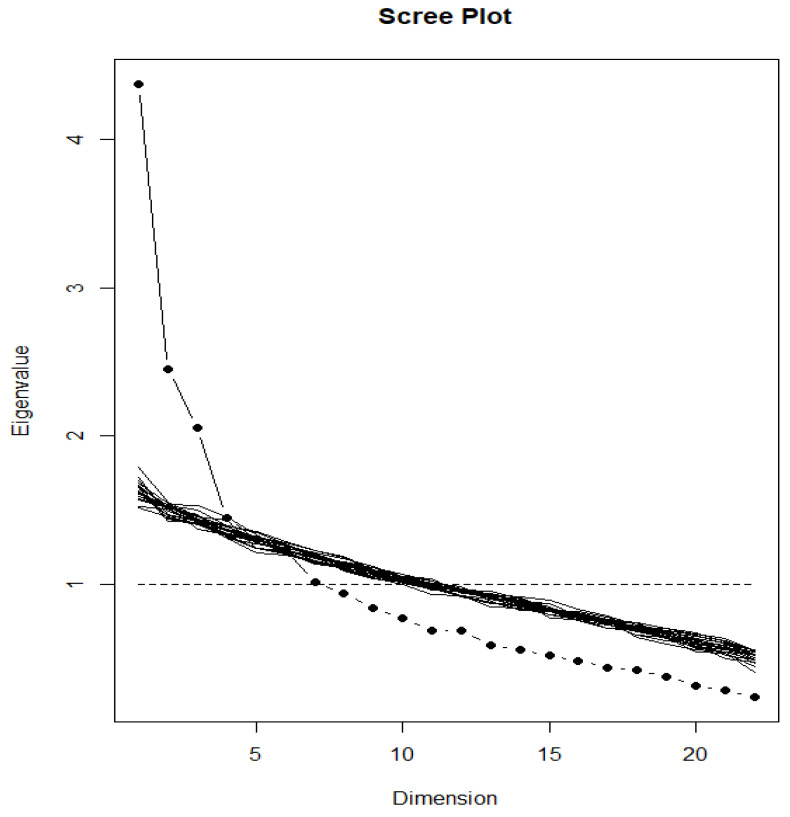
Scree plot of the items in the Food-Mood Questionnaire (*n* = 224).

The EFA and its corresponding scree plot were used to identify the components/constructs of FMQ. Following the parallel analysis approach, a total of twenty scree plots were generated and added to Figure 1 based on twenty randomly generated datasets similar in size to our data. An examination of Figure 1 suggested that three eigenvalues are above “what could be expected by chance”. These results indicate that the Arabic FMQ has three constructs/components, which play a significant role in explaining the variance in its twenty-two items.

The suitability of the data for factor analysis was assessed using Bartlett’s Test of Sphericity and the Kaiser–Meyer Olkin value [25]. Bartlett’s test of sphericity was significant (*p* < 0.001), and the KMO value was 0.749, which is above the generally recommended value of 0.6 [26]. The factor loadings of each FMQ item corresponding to the three identified constructs are presented in Table 2. The rotation method used was Varimax with Kaiser Normalization. Questions 17, 18, 19, 20, 21, and 22 have their largest loading on factor 1, labeled “mental distress”. Questions 1, 2, 3, 6, 7, 10, 11, 12, 15 and 16 have their largest loading on factor 2. These questions were about exercise, breakfast, eating whole-grain products, fruits, flaxseeds/nuts, vegetables, beans, and seafood, as well as supplement intake; therefore, factor 2 was assumed to reflect a “prudent diet”. Factor 3 reflected a “Western diet” and included questions 4, 5, 8, 9, 13, and 14 (intake of dairy, caffeine, white bread/rice/pasta, meat/chicken/turkey, fast food, and sugary foods). The three factors explained 40.353% of the total variance, whereby 19.807%, 11.238%, and 9.308% were explained, respectively, by factors 1, 2, and 3. These results were also supported by CFA, which revealed a Chi-square value/df of 2.28 (<5) and root mean squared error of approximation (RMSEA) of 0.076 (<0.08), indicating a good fit and therefore confirming the factor structure of the FMQ [27].

**Figure 2:**
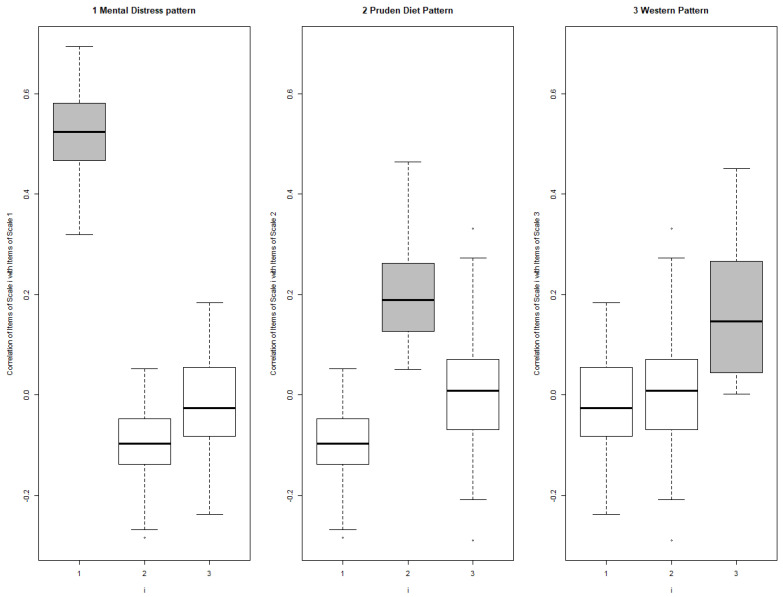
Distribution of inter-item correlations for the Food-Mood Questionnaire subscales (*n* = 224). The gray boxes represent the correlation of the items with other items of their own scale while the white boxes represent the correlations of the items with items from other scales, for scales 1, 2, and 3. This figure shows that for all items, the correlations of items with other items from their own scale are higher than those with items from other scales. A * indicate significant differences at *p* < 0.05.

Summary results of the reliability and internal validity of the FMQ are presented in Table 3. Cronbach’s α was 0.865, 0.716, and 0.531 for the mental distress pattern, prudent diet pattern, and Western diet pattern, respectively, indicating acceptable reliability [28]. A lower reliability was observed for the factors if any item was deleted. The corrected item–total correlation—the correlation between any one item and the total score of the corresponding subscale—reveals that all items are positively correlated with their corresponding subscales. In addition, the distribution of the inter-item correlations for the FMQ shows that the different items correlated better with other items from the same subscale as opposed to the other subscales, further supporting the internal validity of the questionnaire (Figure 2).

For the concurrent validity, the associations between FMQ’s prudent and Western subscales and participants’ diet perceptions were assessed (Table 4). Participants who described their diet as unhealthy (vs. healthy) had a score that was 2.54 units higher for the Western diet subscale (95% CI: 0.81–4.28). The association remained significant after adjustment. The ICC for all items in the questionnaire ranged from 0.667 to 0.866 (all significant with *p*-values < 0.001), indicating moderate to excellent test–retest reliability [29].

## 4. Discussion

The purpose of this study was to validate the Arabic version of the Food-Mood Questionnaire (FMQ) among college students. The findings of the study indicated that the Arabic FMQ is a valid and reliable screening tool to examine the mental health as well as the dietary intake of young adults in the United Arab Emirates.

In this study, the following types of validity were examined: content and face validity, construct validity, concurrent validity, and internal validity. In addition, parallel form reliability and test–retest reliability were investigated.

The content and face validity ensured that the developed questionnaire addressed the mental health and dietary intake of the study population and that the questions used were clear and well understood by the study population. In this study, in addition to the experts’ opinions on the content and face validity, those of the target population (college students) were obtained through the pilot testing of the questionnaire. In their scoping review, Wiering et al. [30] indicated that the active input from the target population or end users in the development stages of an instrument plays a critical role in improving the acceptability, relevance, and quality of the measure and related research.

The results of the EFA examining the construct validity of the FMQ highlighted the three main underlying concepts/dimensions that the questionnaire addressed: mental health and dietary intake (both the prudent and Western patterns). Two previous studies examining the validity of the FMQ in English and Turkish languages also used the EFA. Similar to our results, both studies had a distinct dimension (or factor) related to mental health. However, unlike our results, which showed two dietary patterns, four dietary patterns were derived (healthy pattern, breakfast, Western-like, and supplement) in these studies. The Western pattern described in this study resembles to, a large extent, the Western-like pattern described in the previous investigations. On the other hand, the prudent pattern of this study included the components of the three patterns, healthy, breakfast, and supplement, described in the previous two studies. Arguably, breakfast consumption, as well as supplement use, could be considered part of the prudent dietary pattern, given the overwhelming evidence of an association between breakfast consumption and supplement use and a better dietary pattern [31]. It remains important to note that the number and nature of the factors/dietary patterns resulting from the EFA are usually population-specific and depend largely on the context. In support of the three-factor solution used in this study, a parallel analysis approach with twenty scree plots, generated based on twenty randomly generated datasets that were similar in size to the data, showed that three eigenvalues are above “what could be expected by chance”.

The mental health dimension of the questionnaire represented the six aspects of mental health described in the original Kessler Psychological Distress (K6)-screening scale [32]. They measured the behavioral, cognitive, emotional, and physiological aspects of nonspecific psychological distress based on the diagnostic criteria for major depressive episodes and generalized anxiety disorder. According to Furukawa et al. (2003), the use of polychotomous rather than dichotomous answers to the K6 screening scale, as used in this study, greatly improves the precision of the individual-level predictions of mood and anxiety disorder [33]. Despite its wide use among various age groups and different ethnic populations, the K6 has not yet been translated into Arabic [34]. Therefore, the findings of this study, showing the validity of the K6 as part of the Arabic FMQ, supports the use of this scale in similar populations as a screening tool for psychological distress.

One of the two dimensions of the FMQ related to dietary intake was a prudent pattern associated with frequent intake of flaxseed/nuts, vegetables, fruits, beans, fish, and whole grains. In addition, within this dimension of the questionnaire were questions related to breakfast intake, exercise, and the use of multivitamin supplements. Previous research examining the characteristics of dietary patterns showed that these food items and health behaviors are common denominators of the ‘prudent’ dietary patterns [31]. On the other hand, the Western dietary pattern was characterized by higher intakes of sugary foods, dairy products, meat, fast food, white bread, and caffeine. These foods were also described in previous research to be associated with Western-like patterns. In fact, in the UAE, a study examining dietary patterns revealed two main patterns: a Western pattern, matching the pattern described in this study, and a diverse pattern, which included all the foods of the prudent pattern of this study in addition to mixed traditional dishes [35].

In support of the concurrent validity of the Arabic FMQ were the significant associations between the two derived dietary patterns of the FMQ (prudent and Western) and the corresponding perceived dietary patterns reported by the study participants (healthy and unhealthy). In previous studies, concurrent validity was examined at the same time as the survey, either using questions embedded within the survey or measures obtained from other sources [36]. In this study, we opted for the former option, given the lack of gold standards that would address similar research questions as this study.

The internal reliability as well as the test–retest reliability results were in support of a reliable Arabic version of the FMQ. The three subscales of mental distress pattern, prudent diet pattern, and Western diet pattern produced Cronbach α values of 0.865, 0.716, and 0.531, respectively. There are different reports regarding the acceptable values of alpha, with most indicating an acceptable range between 0.70 and 0.95 [37]. In this study, the low alpha value of the Western dietary patterns could be due to the low number of items in this pattern as compared to the prudent pattern (7 versus 10, respectively). The ICC values used to assess test–retest reliability ranged from 0.667 to 0.866 (all with *p*-values < 0.001), signifying moderate to excellent test–retest reliability.

This study is the first to present a valid and reliable questionnaire that addresses both dietary intake as well as the mental dimensions of health and well-being. Multiple dimensions of validity were examined, including content, face, construct, concurrent, and internal validity. For reliability, both the parallel form and the test–retest reliability were investigated. That said, the findings of this study ought to be considered in light of a few limitations. First, the response rate in this study was low. Low response rates involving students have been frequently encountered in previous studies, and are often attributed to several factors. First, students frequently experience survey fatigue due to the high volume of research requests they receive. Second, their academic, social, and personal commitments can limit the time they have available to participate in studies. Lastly, students may not perceive the relevance or direct benefit of the research to their lives, reducing their motivation to respond. Similar issues have been reported in previous research; for example, Porter and Whitcomb (2005) found that survey fatigue significantly affects response rates among college students and Nulty (2008) highlighted that low response rates are a persistent issue in student feedback surveys [38,39]. Second, the higher participation of females in research as compared to males in this study has also been frequently reported. For instance, a study by Saleh and Bista (2017) found that female graduate students were more likely to respond to online surveys, attributing this to factors such as interest in the survey topic and perceived relevance [40]. Similarly, Sax, Gilmartin, and Bryant (2003) reported that female college students demonstrated higher response rates in web-based surveys, suggesting that women may be more inclined to engage in research participation [41]. Third, despite the fact that the sample population included Arabic-speaking college students, both males and females, from various countries (including the UAE and Arab countries from the Levant, as well as from Asia and North Africa), it may not represent all Arabic-speaking young adults. It is postulated that the methods used for subject recruitment in this study resulted in a convenient sample, whereby the distribution of subjects across many variables, such as gender and nationality, may not be representative of the target population. In light of the aforementioned limitations and in line with the main study purpose, the manuscript does not intend to provide an insight into the dietary patterns or dietary preferences of the Arab population. Further studies using a planned sample would lead to a more representative sample and would therefore enhance the generalizability of the findings.

Lastly, the Arabic FMQ aims to assess the non-specific psychological distress aspect of mental health only. Other important aspects of mental health, such as emotional and social wellbeing, are not addressed. In addition, although the K6 part of the Arabic FMQ is best able to detect mood and anxiety disorders in adolescents, caution is warranted in using it among adolescents with behavior disorders [42].

## 5. Conclusions

The findings of this study showed that the Arabic version of FMQ has acceptable psychometric indicators, reflecting good validity and reliability. The questionnaire presents a concise screening tool for food and mood among college students in the Arab world. The tool could be used to identify gaps and opportunities for improving mental health as well as dietary intake in this population group. The brevity of this validated questionnaire may result in better response rates and a better quality of data compared to the extensive traditional dietary intake methods (such as food frequency questionnaires) or mental health tools. In addition, given the fact that this tool is being translated and validated into other languages, comparative studies across nations could be conducted to better understand the ecological differences in food in relation to mood in different cultures. Nonetheless, it is important to consider the fact that, as a gold standard to measure food and mood is lacking, the relative validity of the questionnaire could not be examined.

## Figures and Tables

**Table 1:** Baseline characteristics of the study population (*n* = 224).

	Frequency	Percent
Age (Mean ± SD)	20.7 ± 4.1	
Gender		
Male	61	27.2
Female	163	72.8
Marital Status		
Ever married	23	10.3
Single	201	89.7
Nationality		
Emirati	137	61.2
Arab (non-Emirati)	79	35.3
Asian/African (Arabic-speaking)	8	3.6
Emirate of Residence		
Sharjah	74	33.0
Other Emirates	150	67.0
Area of Study		
Medicine and Health	98	43.8
Sciences	91	40.6
Engineering	25	11.2
Arts and Humanities	10	4.5
Academic Year		
Foundation year and year 1	80	35.7
Years 2 and 3	106	47.3
Year 4+	38	17.0
Family Income		
Less than AED 10,000	80	35.7
More than AED 10,000	144	64.3
Smoking status		
Yes	36	16.1
No	188	83.9
Dietary pattern		
Healthy (rich in fruits, vegetables, and whole grains)	34	15.2
Unhealthy (rich in refined grains, red meat, and solid fats)	190	84.8

**Table 2:** Factor loadings as derived from the explanatory factor analysis (*n* = 224).

Questions		Factor 1 Mental Distress Pattern	Factor 2 Prudent Diet Pattern	Factor 3 Western Diet Pattern
Q 18	During the past month, about how often did you feel **hopeless**?	0.830	−0.114	
Q 17	During the past month, about how often did you feel so **depressed** that 2thing could cheer up?	0.819		−0.181
Q 22	During the past month, about how often did you feel **restless or fidgety**?	0.765		0.128
Q 20	During the past month, about how often did you feel **nervous**?	0.741		0.131
Q 19	During the past month, about how often did you feel **worthless**?	0.708	−0.132	−0.197
Q 21	During the past month, about how often did you feel that everything was an **effort**?	0.686	−0.158	
Q 7	On weekly average, how many times a day do you consume **flaxseed and/or nuts**?		0.708	
Q 10	On weekly average, how many times do you eat dark green leafy **vegetables** (such as spinach, mlukhiyeh, Swiss chard, kale)?		0.586	
Q 6	On weekly average, how many times a day do you consume **fruits**?	−0.155	0.582	
Q 1	On weekly average, how many times do you **exercise** for at least 20 min? (cardiovascular and strength exercises such as walking, aerobics, gym, weightlifting, etc.)	−0.231	0.534	−0.315
Q 2	In the past 7 days, how many times did you eat **breakfast**?	−0.207	0.531	0.168
Q 11	On weekly average, how many times do you eat **beans** (such as broad beans, fava bean, lentils, chickpeas)?		0.530	
Q 12	On weekly average, how many times do you eat **fish and shellfish** (including sardines and tuna)?		0.487	0.113
Q 16	On weekly average, how many times do you take **fish oil supplements**?		0.454	
Q 15	On weekly average, how many times do you take **multivitamin supplements**?		0.440	
Q 3	On weekly average, how many times do you eat **whole grain products** (such as whole wheat bread, toast, burghul)?	−0.113	0.375	
Q 14	On weekly average, how many times did you consume **sugary foods** such as candy, chocolate, or sweets?	0.142		0.710
Q 4	On weekly average, how many times do you have **dairy** products?	−0.148	0.326	0.638
Q 9	On weekly average, how times do you eat **meat, chicken, or turkey**?			0.576
Q 13	On weekly average, how many times do you eat **fast foods** or, ready-made meals?	0.161	−0.265	0.545
Q 8	On weekly average, how many times do you eat **white bread, rice, and or pasta**?	−0.140	−0.112	0.382
Q 5	On weekly average, how many times do you consume **caffeine** containing food sources (such as coffee, tea, dark chocolate, energy drinks, sodas)?		0.171	0.297
Eigenvalue		4.357	2.472	2.048
% Variance		19.807	11.238	9.308

Extraction method: principal component analysis. Rotation method: Varimax with Kaiser normalization.

**Table 3:** Internal validity of the FMQ scales and their corresponding items. (*n* = 224).

		Scale Mean if Item Deleted	Scale Variance if Item Deleted	Corrected Item Total Correlation	Squared Multiple Correlation	Cronbach’s Alpha if Item Deleted
Mental distress pattern						
Q 18	Hopeless	10.375	20.540	0.770	0.610	0.823
Q 17	Depressed	10.759	19.977	0.750	0.584	0.826
Q 22	Restless or fidgety	9.982	22.349	0.647	0.460	0.846
Q 20	Nervous	9.777	23.026	0.599	0.447	0.853
Q 19	Worthless	11.049	20.513	0.618	0.464	0.853
Q 21	Effort	9.911	21.660	0.605	0.373	0.852
Prudent diet pattern						
Q 7	Flaxseed and/or nuts	18.714	58.070	0.510	0.351	0.674
Q 10	Vegetables	18.098	59.416	0.417	0.246	0.688
Q 6	Fruits	17.500	58.987	0.420	0.252	0.687
Q 1	Exercise	18.188	57.059	0.415	0.242	0.687
Q 2	Breakfast	16.790	55.144	0.387	0.179	0.696
Q 11	Beans	18.580	62.747	0.361	0.217	0.698
Q 12	Fish and shellfish	18.799	64.036	0.337	0.187	0.702
Q 16	Fish oil supplements	19.571	62.354	0.338	0.258	0.700
Q 15	Multivitamin supplements	18.857	57.818	0.349	0.277	0.701
Q 3	Whole grain products	17.835	61.125	0.284	0.113	0.710
Western diet pattern						
Q 14	Sugary foods	16.362	16.097	0.385	0.308	0.430
Q 4	Dairy products	15.446	16.239	0.395	0.235	0.426
Q 9	Meat, chicken, or turkey	14.982	17.560	0.357	0.165	0.453
Q 13	Fast foods	16.844	18.106	0.237	0.231	0.505
Q 8	White bread, rice, and or pasta	15.272	18.405	0.206	0.104	0.519
Q 5	Caffeine	15.268	18.574	0.128	0.072	0.565

Cronbach alpha for the full scale = 0.553; for the mental distress pattern = 0.865; for the prudent diet pattern = 0.716; for the Western diet pattern = 0.531.

**Table 4:** Association between the FMQ dietary patterns (prudent and western) with participants’ perceived dietary patterns. (*n* = 224).

	Prudent Diet Subscale		Western Diet Subscale	
	Crude	Adjusted ˠ	Crude	Adjusted ˠ
Perceived dietary patterns				
Healthy	Ref	Ref	Ref	Ref
Unhealthy	−2.08 (−5.17; 1.02)	−2.28 (−5.37; 0.81)	2.54 (0.81; 4.28)	2.43 (0.67; 4.19)

Values in this table represent β (95%CI), as derived from simple and multiple linear regression analyses. ˠ Adjusted for age, gender and marital status.

## Data Availability

The raw data supporting the conclusions of this article will be made available by the authors on request.

## Supplementary Materials

The following supporting information can be downloaded at: https://www.mdpi.com/article/10.3390/ijerph21111509/s1, Supplementary File: Food and mood questionnaire.
