# Supplementary material for: Validity and Reliability of the Transcultural Arabic Adaptation of the Food-Mood Questionnaire Among College Students
Source: Int J Environ Res Public Health. 2024 Nov 13;21(11):1509. doi: 10.3390/ijerph21111509 (PMC11594256; doi:10.3390/ijerph21111509)
Supplement: Supplementary file 1 [file ijerph-21-01509-s001.zip › ijerph-3133978-supplementary.pdf]

Food and Mood Questionnaire

Section A: Demographics and General Questions

1. What is your gender?
   - Male
   - Female

2. What is your age(years)?
   - ____________

3. What is your marital status?
   - Ever married
   - Single

4. What is your nationality?
   - Emirati
   - Arab (non-Emirati)
   - Asian/African (Arabic-speaking)

5. In which Emirate are you currently living ?
   - Abu Dhabi
   - Dubai
   - Sharjah
   - Ajman
   - Umm Al Quwain
   - Ras Al Khaimah
   - Fujairah

6. What is your major/or professional degree?
   - Medicine and health
   - Sciences
   - Engineering
   - Arts and humanities

7. What is your academic year ?
   - Foundation
   - First year
   - Second year

- Third year
- Fourth year
- Postgraduate

8. What is your income?
   - Less than 10000 AED
   - More than 10000 AED

Section B: lifestyle and addressed smoking (smoker, non-smoker) and dietary patterns

9. Are you a current smoker (shisha,cigarets,vaping,etc..)?
   - Yes
   - No

10. How do you perceive your dietary pattern to be? (Please check the closest answer):
    - Healthy (rich in fruits, vegetables, and whole grains)
    - Unhealthy (rich in refined grains, red meat, and solid fats)

11. On average week, how many times do you exercise at least 20 minutes? (cardiovascular and strength exercise) *Such as walking , aerobic , gym,weightlifting, etc …..
    - None
    - 1 time
    - 2 times
    - 3 times
    - 4 times
    - 5 times or more

12. In the past 7 days, how many times did you eat breakfast?
    - Never
    - 1 time
    - 2 times
    - 3 times
    - 4 times
    - 5 times
    - 6 or 7 times
13. On an average week, how many times do you eat whole grain products? Such as whole wheat bread, toast,burghul,jareesh,)
    - None
    - 1 time
    - 2 times

- 3 times
- 4 times
- 5 times or more

14. On an average week, how many times do you have dairy products?
    - None
    - 1 time
    - 2 times
    - 3 times
    - 4 times
    - 5 times or more

15. On an average week, how many times do you consume caffeine containing food sources ? such as coffee, tea, dark chocolate, energy drinks, sodas
    - None
    - 1 time
    - 2 times
    - 3 times
    - 4 times
    - 5 times or more

16. On an average week, how many times a day do you consume fruits?
    - None
    - 1 time
    - 2 times
    - 3 times
    - 4 times
    - 5 times or more

17. On an average week, how many times a day do you consume flaxseed, and or nuts?
    - None
    - 1 time
    - 2 times
    - 3 times
    - 4 times
    - 5 times or more

18. On an average week, how many times do you eat white bread, rice, and or pasta?
    - None

- 1 time
- 2 times
- 3 times
- 4 times
- 5 times or more

19. On an average week, how times do you eat meat, chicken, or turkey?
    - None
    - 1 time
    - 2 times
    - 3 times
    - 4 times
    - 5 times or more

20. On an average week, how many times do you eat dark green leafy vegetables? Such as Spinach, mlukhiyeh, Swiss chard(silk),Kale …
    - None
    - 1 times
    - 2 times
    - 3 times
    - 4 times
    - 5 times or more

21. On an average week, how many times do you eat beans? such as broad beans, fava bean, lentils, chickpeas…
    - None
    - 1 time
    - 2 times
    - 3 times
    - 4 times
    - 5 times or more

22. On an average week, how many times do you eat fish and shellfish (including sardines and tuna)?
    - None
    - 1 time
    - 2 times
    - 3 times
    - 4 times

- 5 times or more

23. On an average week, how many times do you eat fast foods or, ready made meals?
    - None
    - 1 time
    - 2 times
    - 3 times
    - 4 times
    - 5 times or more

24. On an average week, how many times did you consume sugary foods such as candy, chocolate, or sweets?
    - None
    - 1 time
    - 2 times
    - 3 times
    - 4 times
    - 5 times or more

25. On an average week, how many times do you take multivitamin supplements?
    - None
    - 1 time
    - 2 times
    - 3 times
    - 4 times
    - 5 times or more

26. On an average week, how many times do you take fish oil supplements?
    - None
    - 1 time
    - 2 times
    - 3 times
    - 4 times
    - 5 times or more

Section C: Mental health

27. During the past 12 months, about how often did you feel NERVOUS?
    - None of the time

- A little of the time
- Some of the time
- Most of the time
- All the time

28. During the past 12 months, about how often did you feel HOPELESS?
    - None of the time
    - A little of the time
    - Some of the time
    - Most of the time
    - All the time

29. During the past 12 months, about how often did you feel RESTLESS or FIDGETY?
    - None of the time
    - A little of the time
    - Some of the time
    - Most of the time
    - All the time
30. During the past 12 months, about how often did you feel SO DEPRESSED THAT NOTHING COULD CHEER YOU UP?
    - None of the time
    - A little of the time
    - Some of the time
    - Most of the time
    - All the time

31. During the past 12 months, about how often did you feel THAT EVERYTHING WAS AN EFFORT?
    - None of the time
    - A little of the time
    - Some of the time
    - Most of the time
    - All the time

32. During the past 12 months, about how often did you feel WORTHLESS?
    - None of the time
    - A little of the time
    - Some of the time
    - Most of the time

- All the time
